# Pharmacokinetics and Alterations in Glucose and Insulin Levels After a Single Dose of Canagliflozin in Healthy Icelandic Horses

**DOI:** 10.1111/jvp.13476

**Published:** 2024-08-07

**Authors:** Peter Michanek, Johan Bröjer, Inger Lilliehöök, Cathrine T. Fjordbakk, Minerva Löwgren, Mikael Hedeland, Jonas Bergquist, Carl Ekstrand

**Affiliations:** ^1^ Department of Animal Biosciences Swedish University of Agricultural Sciences Uppsala Sweden; ^2^ Department of Clinical Sciences Swedish University of Agricultural Sciences Uppsala Sweden; ^3^ Department of Companion Animal Clinical Sciences Norwegian University of Life Sciences Oslo Norway; ^4^ Department of Medicinal Chemistry Uppsala University Uppsala Sweden; ^5^ Department of Chemistry‐BMC Uppsala University Uppsala Sweden

**Keywords:** canagliflozin, equine metabolic syndrome, graded glucose infusion, pharmacokinetics, SGLT2 inhibitor

## Abstract

Canagliflozin (CFZ) is a sodium‐glucose cotransporter‐2 inhibitor that has shown promising results as a drug for the treatment of insulin dysregulation in horses. Even though CFZ is used clinically, no pharmacokinetic data has previously been published. In this study, the pharmacokinetics of CFZ after administration of a single oral dose of 1.8 mg/kg in eight healthy Icelandic horses was examined. Additionally, the effect of treatment on glucose and insulin levels in response to a graded glucose infusion was investigated. Plasma samples for CFZ quantification were taken at 0, 0.33, 0.66, 1, 1.33, 1.66, 2, 2.33, 2.66, 3, 3.5, 4, 5, 6, 8, 12, 24, 32, and 48 h post administration. CFZ was quantified using UHPLC coupled to tandem quadrupole mass spectrometry (UHPLC‐MS/MS). A non‐compartmental analysis revealed key pharmacokinetic parameters, including a median *T*_max_ of 7 h, a *C*_max_ of 2350 ng/mL, and a *t*_1/2Z_ of 28.5 h. CFZ treatment reduced glucose (AUC_GLU_, *p* = 0.001) and insulin (AUC_INS_, *p* = 0.04) response to a graded glucose infusion administered 5 h after treatment. This indicates a rapid onset of action following a single dose in healthy Icelandic horses. No obvious adverse effects related to the treatment were observed.

## 1 Introduction

Insulin dysregulation (ID) is a consistent feature of equine metabolic syndrome, often presenting with hyperinsulinemia as a common occurrence. Increased risk of laminitis in horses and ponies with ID is well known, and hyperinsulinemia has been experimentally shown to induce this condition (de Laat et al. 2010; Asplin et al. 2007). The cornerstone in the management of ID involves dietary restrictions and exercise (Durham et al. 2019). Exercise is beneficial in horses with ID by increasing insulin sensitivity (Stewart‐Hunt, Geor, and Mccutcheon 2006), but in cases with acute laminitis exercise should be avoided to not exacerbate the damage to the lamellar tissue of the hoof (Durham et al. 2019). In horses with ID, it is recommended to feed a forage low in nonstructural carbohydrates (NSC) to decrease postprandial insulin levels (Macon et al. 2022, 2023). Additionally, in overweight individuals, weight reduction through energy restriction is also recommended, as this has been shown to increase insulin sensitivity (Van Weyenberg et al. 2008). However, the recommended management changes are not always sufficient in preventing laminitis (Kellon and Gustafson 2023; Sundra, Kelty, and Rendle 2023) and laminitis in affected horses is a common cause of euthanasia (Luthersson et al. 2017).

Pharmacological therapy is sometimes used in conjunction with management for the treatment of refractory cases of ID. Metformin has been used due to a potentially positive impact on ID in horses. The existing body of literature, nevertheless, presents conflicting findings, leading to a concern about the true efficacy of metformin in treating ID in equines (Colmer et al. 2023; Rendle et al. 2013; Durham, Rendle, and Newton 2008; Vick et al. 2006). This concern is further compounded by the observation of notably low bioavailability of the drug (Hustace, Firshman, and Mata 2009; Tinworth et al. 2010). Levothyroxine, occasionally employed in the treatment of ID, exhibits potential in increasing insulin sensitivity (Frank, Elliott, and Boston 2008; Sommardahl et al. 2005) and has been linked to increased weight loss in horses (Frank, Elliott, and Boston 2008; Sommardahl et al. 2005; Chameroy, Frank, and Elliott 2010). Yet, there is still uncertainty surrounding its efficacy in ID management (Chameroy, Frank, and Elliott 2010).

Sodium‐glucose cotransporter (SGLT) 2 inhibitors (SGLT2i) is a recently discovered drug class introduced for the treatment of type II diabetes in humans about a decade ago (Haas et al. 2014). SGLT2 is a protein located in the proximal tubule of the nephron where it accounts for the majority of the glucose reabsorption. By inhibiting the SGLT2 protein, SGLT2i increases urinary glucose excretion, leading to lower glucose levels in the blood. Since elevated blood glucose is the most important stimulus for insulin secretion, this mechanism of action leads to the hypothesis that SGLT2i could be beneficial in treating horses with ID, by lowering hyperinsulinemia and subsequently preventing laminitis. Three different SGLT2i have been used for treating ID in horses, namely canagliflozin (CFZ) (Kellon and Gustafson 2022, 2023; Lindase et al. 2023), ertugliflozin (Kellon and Gustafson 2023; Sundra, Kelty, and Rendle 2023), and velagliflozin (Meier et al. 2018, 2019). All have shown encouraging results in lowering insulin levels and preventing laminitis. However, no SGLT2i are registered for use in horses.

CFZ is an SGLT2i registered for the treatment of type II diabetes in humans and has been shown to increase glycemic control, promote weight loss, and reduce the risk of cardiovascular‐ and renal events in human patients (ElSayed et al. 2022). Initial observations from two case series have shown encouraging results in reducing both insulin levels and laminitis occurrence in hyperinsulinemic equine patients after daily dosing of 0.3–0.6 mg/kg CFZ (Kellon and Gustafson 2022, 2023). Additionally, a recent randomized, double‐blind, and placebo‐controlled clinical trial showed that administering 0.6 mg/kg CFZ daily for 4 weeks was highly effective in reducing postprandial hyperinsulinemia and beta cell responsiveness, in response to an oral sugar test in ID horses (Lindase et al. 2023). To the authors' knowledge, there are no published pharmacokinetic studies of any SGLT2i in horses, nor studies on their effects in healthy horses. The main aim of the present study was to explore the concentration–time course following oral CFZ administration to healthy Icelandic horses. Additionally, secondary aims were to investigate glucose dynamics, biochemical changes along with monitoring for potential adverse effects associated with CFZ treatment in healthy Icelandic horses.

## 2 Materials and Methods

### 2.1 Horses

Eight adult Icelandic horses were included in the study. All horses were considered healthy based on history and clinical examination. The horse's average body weight was 395 kg (range, 357–436) and age 15 years (range, 11–23). The median Body Condition Score (Henneke et al. 1983) was 6 (range, 6–7) and Cresty Neck Score (Carter et al. 2009) 2 (range, 2–3). Throughout the year, the horses mainly reside outside in loose housing conditions and are provided grass haylage or access to pasture, along with unrestricted access to water. Horses were regularly ridden, predominantly several times a week as part of their routine as riding school horses. During the study period, however, the horses' routine was modified: they were stabled at night and during experimental interventions. Outside these times, they resided in their usual outdoor environment.

### 2.2 Experimental Design

**FIGURE 1:**
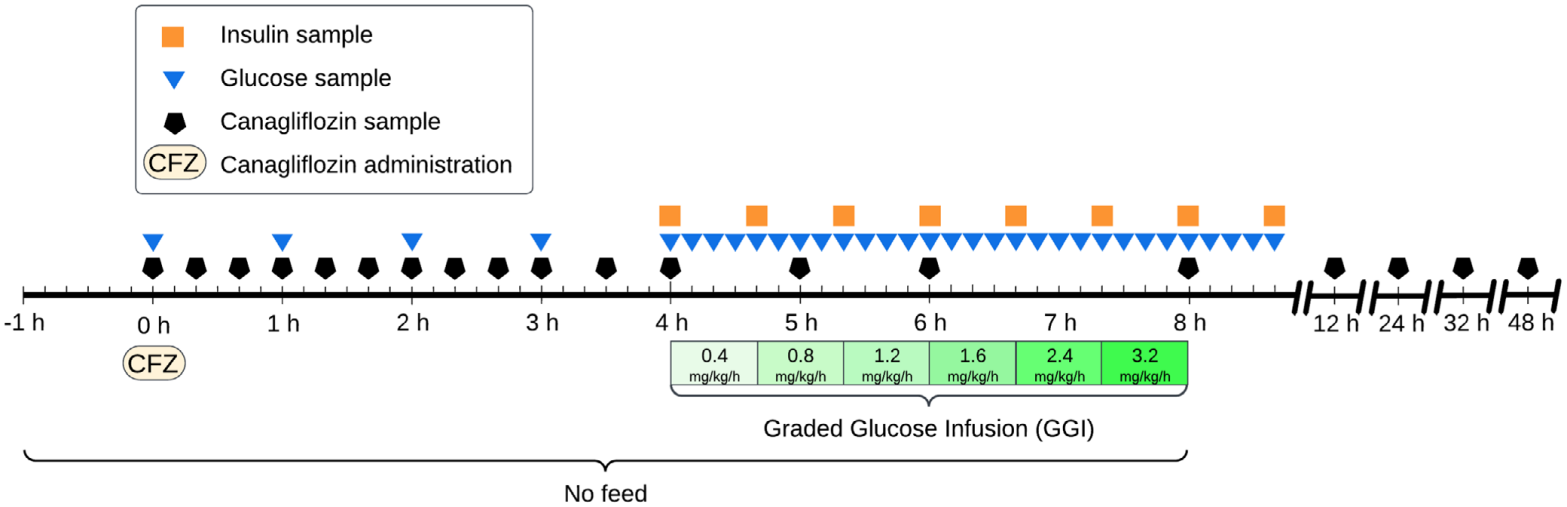
Experimental timeline for canagliflozin treatment. A parallel timeline for the placebo treatment was implemented, with the only difference being the absence of drug administration and the lack of sampling at the final four time points.

The study was designed as a non‐blinded, two‐treatment crossover study, where horses initially were subjected to placebo treatment followed by a single oral dose of CFZ (Invokana, 100 mg, Berlin‐Chemie AG, Berlin, Germany) after a 48‐h washout period. The target dose was 1.8 mg/kg; however, due to the limitations of tablet sizes, the exact doses administered deviated by a small amount, as detailed in Table 1. An overview of the experimental timeline for CFZ is presented in Figure 1. Feed was withheld from the horses for a period of 9 h, beginning 1 h before the administration of either the drug or placebo and concluding with the completion of the graded glucose infusion (GGI). The drug was administered in one deciliter of pre‐soaked alfalfa pellets, whereas the placebo treatment consisted of one deciliter pre‐soaked alfalfa pellets without any additive. The day before the placebo treatment, an indwelling catheter was aseptically placed in each jugular vein. Bilateral indwelling catheters were necessitated by the need for simultaneous infusion and sampling. Prior to placing the catheter, the skin was desensitized using topical local anesthetic cream containing lidocaine and prilocaine (EMLA, 25 mg/g + 25 mg/g, Aspen Nordic, Ballerup, Denmark). The Animal Ethics Committee in Uppsala, Sweden, granted ethical approval for the study (5.8.18‐05506/2022).

**TABLE 1:** Individual and summarized statistics for pharmacokinetic parameters obtained through non‐compartmental analysis, following the administration of a 1.8 mg/kg oral dose of canagliflozin to a group of eight horses.

ID	Dose (mg·kg^−1^)	*C*_max_ (ng·mL^−1^)	AUC_0‐48h_ (h·ng·mL^−1^)	AUC_0‐inf_ (h·ng·mL^−1^)	*t*_1/2_ (h)	*k*_el_ (h^−1^)	*T*_max_ (h)	CL/F (mL·h^−1^·kg^−1^)
1	1.83	3417	67,030	113,732	39.1	0.018	3.5	16.1
2	1.87	2599	58,790	88,466	29.1	0.024	5	21.1
3	1.89	2334	57,917	87,959	29.6	0.023	8	21.5
4	1.91	4049	58,004	79,265	25.1	0.028	4	24.2
5	1.88	1723	53,965	98,490	42.1	0.016	8	19.1
6	1.91	1630	45,818	72,057	28.0	0.025	8	26.6
7	1.67	2366	46,458	63,776	23.2	0.030	6	26.3
8	1.68	871	29,429	46,238	27.3	0.025	12	36.4
Median	1.87	2350	55,941	83,612	28.5	0.024	7	23.8
Geometric mean	1.83	2166	50,857	78,676	29.8	0.023	6.3	23.3
Geometric SD	1.06	1.62	1.29	1.32	1.23	1.23	1.51	1.28

Abbreviations: AUC_0–48h_, the area under the curve from the time of administration to the last observation at 48h post administration; AUC_0–inf_, the sum of AUC_0–48h_ and its extrapolation to infinity; CL/F, the ratio between total clearance and bioavailability; *C*_max_, peak plasma concentration; *k*_el_, elimination rate constant; *t*_1/2Z_, terminal half‐life; *T*_max_, time of maximum concentration.

### 2.3 Graded Glucose Infusion

Following a 5‐h fasting period and 4 h after administering CFZ or placebo, a GGI (Byrne, Sturis, and Polonsky 1995), adapted for use in horses, was initiated. Glucose (Glucos Fresenius Kabi 300 mg/mL, Fresenius Kabi, Uppsala, Sweden) was infused using an Infusomat Space (B. Braun Melsungen AG, Melsungen, Germany), at incremental rates of 0.4, 0.8, 1.2, 1.6, 2.4, and 3.2 mg/kg/min, with each infusion rate maintained for a 40‐min duration.

### 2.4 Sampling

Sample collection was primarily conducted through indwelling jugular vein catheters, except for the final three samples of the CFZ treatment, which were obtained via direct venipuncture. Subsequently, the blood was transferred into evacuated tubes, with EDTA 2K for plasma and clot activator for serum. While the tubes with EDTA 2K were centrifuged immediately post sampling, the tubes for serum were first allowed to clot at room temperature for 40 min. Both plasma and serum samples were centrifuged at 1950*g* for 10 min. The supernatant was transferred and immediately frozen at −20°C and within a week relocated to −80°C pending analysis. Plasma for CFZ analyses were collected at 0, 0.33, 0.66, 1, 1.33, 1.66, 2, 2.33, 2.66, 3, 3.5, 4, 5, 6, 8, 12, 24, 32, and 48 h post administration. Plasma for glucose analysis was sampled at 0, 1, 2, 3, and 4 h post administration and then every 10 min beginning at the start of the GGI and ending at 40 min after GGI completion. Plasma for insulin analysis was sampled at the GGI start and every 40 min for the entire infusion and until 40 min after completion. Serum was sampled exclusively in the evening at three time points. The first sampling occurred the evening before any intervention, and subsequent samplings were 32 h after administration of each respective treatment.

### 2.5 Quantification of Canagliflozin

Plasma samples (200 μL of the study sample, calibrator, or QC) were spiked with 100 μL of internal standard solution (750 ng/mL of canagliflozin‐d4). Protein precipitation was carried out with the addition of 400 μL of acetonitrile, where afterward the samples were vortexed for 10 min and centrifuged at 13,000*g* at 4°C for 10 min. The supernatant was evaporated and the residue was reconstituted in 100 μL of methanol.

Quantitative analysis of CFZ was carried out with ultra high‐performance liquid chromatography (UHPLC) coupled to tandem quadrupole mass spectrometry via an electrospray interface operating in the positive mode. The method was partly validated according to the ICH/EMA guidelines. The instrumentation consisted of a Shimadzu Nexera UHPLC/SFC system (Shimadzu Corporation, Kyoto, Japan) for high‐efficiency liquid chromatographic separation, coupled to a Shimadzu LC MS‐8060NX tandem quadrupole mass spectrometer. The chromatographic column was a C18 Shim‐Pack Scepter (100 × 3 mm length × inner diameter and a particle diameter of 2.1 μm) from Shimadzu Corporation. The mobile phase consisted of water:methanol (15:85 v/v) with 0.1% formic acid delivered isocratically.

The mass spectrometric detection was performed in the Selective Reaction Monitoring (SRM) mode with the following transitions: canagliflozin [M+H]^+^ 462.1 > 191.1 (collision energy: 25 V) and canagliflozin‐d4 [M+H]^+^ 466.1 > 267.0 (collision energy: 15 V). Calibration curves were constructed by linear regression of the peak area ratio (analyte/internal standard) as a function of the analyte concentration in the range of 20–10,000 ng/mL CFZ in plasma. Quality control (QC) samples were analyzed at concentration levels 30, 75, 3000, 6000, and 10,000 ng/mL. The between‐run accuracy was in the range of 98%–101% and the between‐run precision was 0.99%–1.58% (RSD).

### 2.6 Biochemistry and Insulin

Plasma glucose concentrations were determined using a YSI 2500 Glucose/Lactate Analyzer (YSI Incorporated, Yellow Spring, Ohio). Plasma insulin concentrations were quantified using the Mercodia Equine Insulin ELISA (Mercodia, Uppsala, Sweden), which has been previously validated for use in equines (Öberg et al. 2012). All samples from each individual were analyzed in duplicate on the same ELISA plate, with the mean value of the duplicates used for each sample. Concentration accuracy was verified using high and low controls included in each assay run. The intra‐assay coefficient of variation for the insulin ELISA was calculated using the root mean square approach. Serum samples were batch analyzed for albumin, chloride, creatinine, gamma‐glutamyl transferase (GGT), glutamate dehydrogenase (GLDH), potassium, serum amyloid A (SAA), sodium, total protein, and triglycerides using an automatic biochemistry analyzer, DxC 700AU (Beckman Coulter, California, USA) with reagents from Beckman Coulter, except SAA (Gentian Diagnostics, Moss Norway) and GLDH (Roche Diagnostics, Rotkreuz, Switzerland.).

### 2.7 Data Analysis

CFZ plasma data were analyzed non‐compartmentally. The analysis was carried out using PKanalix™ 2023R1 (Lixoft SAS, a Simulations Plus company, Antony, France). For the calculation of the rate constant of the terminal disposition phase (*λ*_z_), uniform weighting was used, and point selection was based on adjusted *R* ^2^ values. The area under the curve (AUC) was estimated through linear‐up log‐down method. Concentrations below the lower limit of quantification occurring prior to the time of maximum concentration (*T*_max_) were set to zero. The estimated parameters included *T*_max_, peak plasma concentration (*C*_max_), elimination rate constant (*k*_el_), terminal half‐life (*t*_1/2Z_), and the ratio between total clearance and bioavailability (CL/F). Additionally, the analysis determined the area under the concentration–time curve from the time of administration to the last observed point at 48 h (AUC_0–48h_), as well as the sum of AUC_0–48h_ and its extrapolation to infinity (AUC_0–inf_).

The effect of the treatment on glucose and insulin levels in response to the GGI was evaluated using several parameters. These include the area under the curve for glucose (AUC_GLU_), the area under the curve for insulin (AUC_INS_), the peak glucose concentration (Max_GLU_), the peak insulin concentration (Max_INS_), the incremental area under the curve for glucose (iAUC_GLU_), and the incremental area under the curve for insulin (iAUC_INS_). Additionally, two indices were calculated: the ratio of iAUC_INS_ to iAUC_GLU_ (iAUC_INS_/iAUC_GLU_) and the change in insulin relative to the change in glucose (Δinsulin/Δglucose). The iAUC_INS_/iAUC_GLU_ and Δinsulin/Δglucose (Lin et al. 2016) are measures of beta cell responsiveness. All parameters related to glucose metabolism and beta cell responsiveness, except for the Δinsulin/Δglucose ratio, were calculated for the entire duration of the GGI and the 40 min following the completion of the infusion. The Δinsulin/Δglucose ratio was calculated only for the duration of the GGI.

All statistical computations were conducted using R version 4.3.1 (R Core Team 2023). For calculating the AUC_GLU_ and AUC_INS_, the trapezoidal method was employed. The iAUC_GLU_ and iAUC_INS_ were derived similarly, but with the baseline concentration of either parameter at the infusion's onset subtracted. The Δinsulin/Δglucose parameter and its associated *r* ^2^‐value for each horse were determined by fitting a least squares regression line. The treatment effects on biochemistry parameters were calculated based on the change from baseline to post‐treatment for each intervention. The first sample was utilized as the baseline for the placebo run, whereas the second sample functioned both as the post‐placebo measurement and as the baseline for the CFZ treatment. The third sample represented the post‐treatment measurement for the CFZ intervention.

To assess differences between treatment groups, either the Wilcoxon signed‐rank test or the paired t‐test was used, based on an evaluation of data normality conducted through visual inspection of quantile‐quantile plots. When paired differences were normally distributed, a 95% confidence interval (CI) for the difference was also calculated. All tests were two‐sided, and statistical significance was set at an alpha level of 0.05.

## 3 Results

Seven out of the eight horses successfully underwent all interventions. One horse was excluded from the glucose dynamics segment of the trial due to the development of a perivascular hematoma, resulting in the removal of one out of two indwelling catheters. Nevertheless, this horse was able to complete the pharmacokinetic portion of the trial. No adverse effects of the CFZ treatment were observed in any of the horses.

### 3.1 Non‐Compartmental Analysis

**FIGURE 2:**
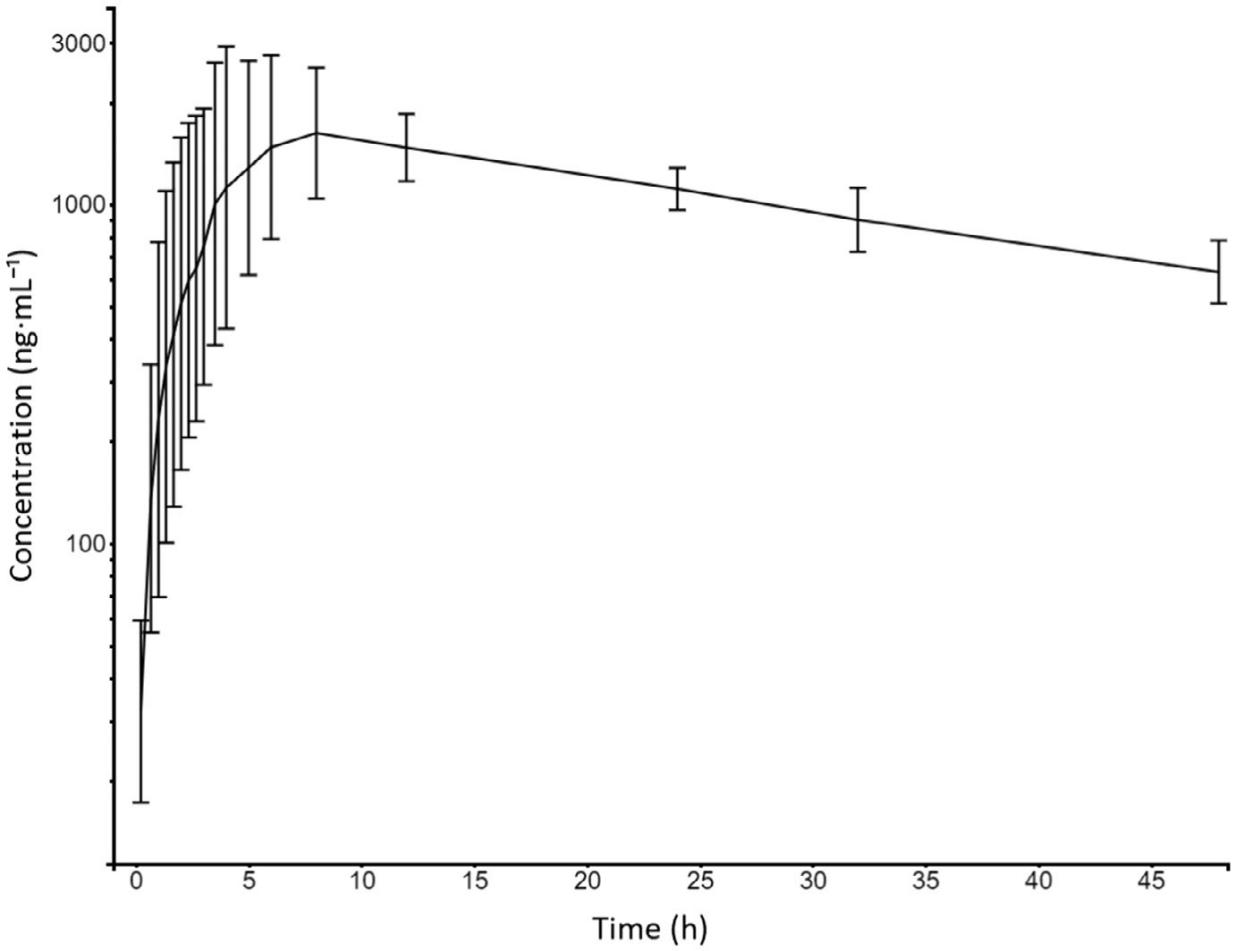
Semi‐logarithmic plot displaying the canagliflozin concentration time‐courses following oral administration of 1.8 mg/kg canagliflozin to eight Icelandic horses. The plot shows the geometric mean ± standard deviation.

Plasma concentrations of CFZ from all horses were below the limit of quantification (LOQ) at 0 h and within the quantification range 1 h post administration. Plasma concentrations remained within the quantification range for the remaining sampling period of 48 h. Figure 2 displays the concentration‐time course of CFZ. Individual estimates of *λ*_z_ were calculated using a minimum of the last three time points, covering a 24‐h period. Additional time points in the terminal phase were included if they improved the adjusted *R* ^2^ value, which had a median of 0.98 (range: 0.95–0.99). Individual and summary statistics from non‐compartmental analysis are summarized in Table 1.

### 3.2 Response to Treatment With CFZ on Glucose, Insulin, and Indexes of Beta Cell Response

**FIGURE 3:**
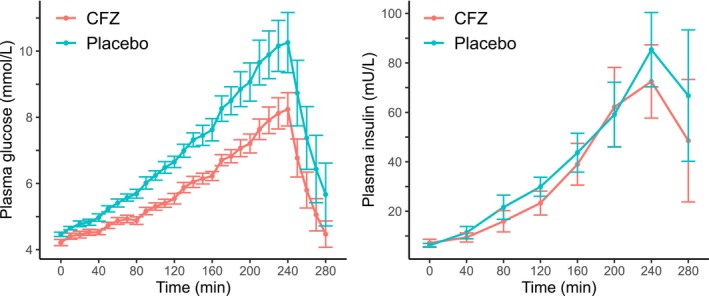
Time‐course profiles of mean plasma glucose (left panel, in mmol/L) and plasma insulin concentrations (right panel, in mU/L) for both placebo and canagliflozin (CFZ) treatments during, and 40 min post a graded glucose infusion in seven Icelandic horses. Error bars represent ± standard error. The infusion commenced at time 0 and concluded at 240 min.

The temporal changes in plasma glucose and insulin concentrations during and for 40 min following the GGI are depicted in Figure 3. Mean ± SD fasting glucose at the start of the infusion (4 h post treatment administration) was 4.5 ± 0.17 and 4.2 ± 0.25 mmol/L for placebo and CFZ, respectively (*p* = 0.03). Treatment with CFZ lowered the total glucose and insulin exposure as measured with AUC (*p* = 0.001 and 0.04, respectively). The incremental AUC for glucose (*p* = 0.006) and insulin (*p* = 0.02), as well as maximum glucose concentrations (*p* = 0.004), were lowered when treated with CFZ. Indices of beta cell responsiveness and maximum insulin concentrations were not statistically different (Table 2). The median *R* ^2^ for Δinsulin/Δglucose was 0.95 (range 0.85–0.98) for CFZ and 0.93 (0.78–0.98) for placebo. The intra‐assay CV for the duplicate analysis of insulin was 4.5%.

**TABLE 2:** Summary statistics of key parameters related to glucose metabolism and beta cell responsiveness, segmented by treatment. For each parameter, the table lists the mean ± SD if the distribution is normal, and the median (range) for non‐normally distributed parameters. Additionally, the table includes the mean differences [95% CI] between treatments for normally distributed differences. *p* Values are also provided to determine the statistical significance of these differences.

Parameter	Treatment	Mean ± SD or Median (IQR)	Mean difference [95% CI]	*p* Value
AUC_GLU_ (min·mmol×L^−1^)	Placebo CFZ	1982 ± 263 1647 ± 138	−334 [−480, −189]	0.001*,^a^
iAUC_GLU_ (min·mmol×L^−1^)	Placebo CFZ	731 ± 280 467 ± 126	−264 [−421, −107]	0.006*,^a^
AUC_INS_ (min·mU·L^−1^)	Placebo CFZ	9641 (8107–12094) 7227 (7930–9577)	−1494 [−2907, −80]	0.04*,^a^
iAUC_INS_ (min·mU·L^−1^)	Placebo CFZ	8155 (6417–10692) 5781(5295–7938)	−1696 [−3033, −360]	0.02*,^a^
Max_GLU_ (mmol·L^−1^)	Placebo CFZ	10.4 ± 2.3 8.3 ± 1.3	−2.1 [−3.2, −0.9]	0.004*,^a^
Max_INS_ (mU·L^−1^)	Placebo CFZ	80.4 (59.3–101.9) 54.3 (52.3–75.0)	−15.1 [−30.9, 0.7]	0.06^a^
Δinsulin/Δglucose (mU·mmol^−1^)	Placebo CFZ	14.3 ± 7.8 17.7 ± 8.5	NA	0.16^b^
iAUC_INS_/iAUC_GLU_ (mU·mmol^−1^)	Placebo CFZ	16.0 (8.9–19.4) 16.0 (10.3–20.3)	NA	0.38^b^

Abbreviations: AUC_GLU_, the area under the curve for glucose; AUC_INS_, the area under the curve for insulin; iAUC_GLU_, the incremental area under the curve for glucose; iAUC_INS_, the incremental area under the curve for glucose; Max_GLU_, peak glucose concentration; Max_INS_, peak insulin concentration; NA, not applicable; Δinsulin/Δglucose, change in insulin relative to the change in glucose.

* *p* < 0.05.

^a^ *p* Value calculated using a paired *t*‐test.

^b^ *p* Value calculated using a Wilcoxon signed‐rank test.

### 3.3 Biochemistry

The impact of placebo and CFZ treatment was statistically different across various biochemical parameters. Despite the variability, the vast majority of measured variable values were within the laboratory's established reference ranges. In contrast, GLDH showed a notable increase (median increase 402%) between the second samples, collected after placebo treatment, and the third samples, collected after the CFZ treatment. However, this increase did not achieve statistical significance. Comprehensive biochemical data, laboratory reference ranges, and associated *p*‐values from Wilcoxon signed‐rank tests are available in Table A1.

## 4 Discussion

This present study is to the best of the authors’ knowledge the first to explore the pharmacokinetics of any SGLT2i in horses, filling a notable knowledge gap in veterinary pharmacology. There was marked inter‐individual variation in the initial phase of the plasma concentration‐time course, whereas the terminal phase displayed lower variability between horses (Figure 2). It is common in pharmacokinetic studies to observe more variation during the absorption phase than during the elimination phase due to several factors (Vinarov et al. 2021). In the present study, the horses were fed just 1 h prior to drug administration, which may have increased the variation in absorption due to differences in gastric emptying times and the amount of content in the stomach. Additionally, because the horses chewed the tablets instead of swallowing them whole as instructed for humans, this chewing potentially increased the variation by causing delays in some cases until the entire tablet content reached the gastrointestinal tract for absorption. Although the elimination phase generally showed lower variability compared to absorption, two horses exhibited significantly longer half‐lives (39 and 42 h, respectively). This observation might be clinically important, and it is possible that there could be even greater variability and associated risks of drug accumulation in diseased populations, which may differ physiologically from the healthy Icelandic horses studied here.

In this population of healthy Icelandic horses, the elimination half‐life (median: 28.5 h) was longer compared to the 10.6–13.1 h seen in healthy human subjects (Devineni and Polidori 2015), and approximately 8 and 13 h in rats (Dong et al. 2018) and dogs (Zhou et al. 2019), respectively. The longer elimination half‐life seen in these horses may offer advantages in terms of dosing frequency, more stable therapeutic levels, and cost‐effectiveness, but may also increase the risk of drug accumulation and potential toxicity. Moreover, if an adverse effects occur, pharmacologically active plasma concentrations will likely be maintained over a longer period of time due to the longer terminal half‐life. The elimination process appears to be consistent with first‐order kinetics. However, to verify first‐order pharmacokinetics, pharmacokinetic studies employing various doses should be conducted. In the absence of comparative intravenous CFZ data, determining bioavailability in this study is not possible. That being said, the observed median *C*_max_ of 2350 ng/mL after oral administration of 1.8 mg/kg in this study suggests a promising bioavailability profile when compared to humans having a mean *C*_max_ of 2504 (±482) ng/mL after receiving on average 3.8 mg/kg and having a 65% bioavailability (Devineni et al. 2015). It is important, however, to acknowledge that *C*_max_ after extravascular administration is influenced by various pharmacokinetic factors besides bioavailability, including distribution, absorption rate, and elimination.

The dose administered in this study was 1.8 mg/kg, which is likely considerably higher than the daily dose needed for treating horses with ID, as doses of 0.3–0.6 mg/kg have been suggested to be clinically effective (Kellon and Gustafson 2022, 2023; Lindase et al. 2023). The decision to use a higher dose of 1.8 mg/kg in this study was guided by its single‐dose design and the aim to examine both tolerability and pharmacokinetic outcomes at elevated plasma concentrations.

In this study, we observed a significant effect on the response to intravenous glucose in healthy Icelandic horses, occurring just hours after a single oral dose of CFZ. The reductions in insulin and glucose levels observed following CFZ treatment are consistent with existing research on SGLT2 inhibitors in ID horses (Kellon and Gustafson 2022, 2023; Sundra, Kelty, and Rendle 2023; Lindase et al. 2023; Meier et al. 2018, 2019). No statistically significant impact was noted on parameters measuring beta cell responsiveness. This aligns with the established mechanism of action for SGLT2i, where the primary effect is increasing urinary glucose excretion, which should not directly affect beta cell responsiveness. However, previous research on ID horses receiving 0.6 mg/kg CFZ daily for 3 weeks, showed a decrease in beta cell responsiveness, as evaluated with an oral sugar test (Lindase et al. 2023). This discrepancy is speculated to be due to the need for an extended treatment period to reveal these changes, and/or the chance that they do not manifest in equines without ID. In a study on healthy human subjects receiving CFZ treatment, a plasma glucose and serum insulin lowering effect was observed in the first meal at 0.5 h post‐treatment, but not in subsequent meals at 4.5 and 10.5 h after treatment (Sha et al. 2011). This pattern implies that CFZ temporarily blocks intestinal glucose absorption, which is proposed to result from the inhibition of SGLT1 due to high local concentrations of the drug (Polidori et al. 2013). The present study in horses, utilizing an intravenous glucose test, indicates that the observed differences in glucose levels are not due to changes in intestinal absorption. However, this does not exclude the possibility that alterations in intestinal absorption could still be contributing to the drug's overall effects in horses. Investigating these potential changes in intestinal absorption and their impact on the efficacy of CFZ could be a topic for future study.

The main limitation of this study is the non‐randomized design where horses received a placebo and then 48 h later received CFZ treatment. This design may lead to biased results, as the effects observed could be influenced by factors other than the treatment. This approach was necessitated by the limited time window available for accessing the horses combined with the desire to prevent any carry‐over effects from the CFZ to the placebo phase. While this design should not compromise the pharmacokinetic evaluation and is unlikely to influence the glucose and insulin dynamics—given these parameters’ very brief time outside physiological levels and rapid return to baseline—it does add complexity to the interpretation of biochemistry results. Various factors, for example changes in activity or environment and frequent blood sampling, could potentially affect the biochemical parameters observed. Although several parameters showed statistically significant differences, the magnitude of these changes was mostly minor and remained within the laboratory's internal reference ranges. Consequently, caution is advised in interpreting these biochemical alterations. Interestingly, the increase in GLDH levels following CFZ administration was conspicuous. While not statistically significant, the finding may have clinical relevance and thus warrants further investigation. Triglycerides, previously reported to increase in horses treated with CFZ (Kellon and Gustafson 2023; Lindase et al. 2023), exhibited a mild elevation in this study. This could perhaps be more pronounced in a multiple‐dosing regimen. It should once again be noted that the slight observed changes in clinical chemistry parameters might also be attributed to limitations in the study design.

## 5 Conclusion

The CFZ concentration‐time course after a 1.8 mg/kg single dose administered per os was characterized by a median *C*_max_, *T*_max_, and *t*_1/2Z_ of 2350 ng/mL, 7 h and 28.5 h, respectively. Treatment with CFZ showed glucose and insulin lowering effects in healthy Icelandic horses as measured with a GGI. No obvious adverse effects related to CFZ were observed. Further research is needed to assess long‐term CFZ treatment and potential risks in insulin dysregulated horses.

## Author Contributions

C. Ekstrand, J. Bröjer, and P. Michanek formulated the study idea and overall concept. J. Bergquist and M. Hedeland developed the assay for quantifying canagliflozin concentrations and authored that section of the manuscript. I. Lilliehöök and P. Michanek conducted the biochemistry data analysis, with P. Michanek performing the glucose and insulin assays. P. Michanek conducted the non‐compartmental and statistical analyses and was the main author of the manuscript. C. Ekstrand, M. Löwgren, and P. Michanek conducted the clinical aspects of the study. All authors participated in refining the study, editing the manuscript, and approved the final version.

## Ethics Statement

All study procedures complied with the application approved by the Animal Research Ethics Committee in Uppsala, Sweden (5.8.18‐05506/2022).

## Conflicts of Interest

The authors declare no conflicts of interest.

## Supporting information


Data S1.

## Data Availability

The data that support the findings of this study are available from the corresponding author (upon reasonable request).
