# Supplementary material for: Pharmacokinetics and Alterations in Glucose and Insulin Levels After a Single Dose of Canagliflozin in Healthy Icelandic Horses
Source: J Vet Pharmacol Ther. 2024 Aug 7;48(Suppl 1):41–9. doi: 10.1111/jvp.13476 (PMC11736998; doi:10.1111/jvp.13476)
Supplement: Supplementary file 1 — Data S1. [file JVP-48-41-s001.docx]

### Supplement

**Table A1.** Median (IQR) of biochemistry parameters from three distinct samples from eight Icelandic horses. The table also show the median change, with percentage change in brackets, following placebo and canagliflozin treatments. The change between the first and second samples is attributed to the placebo run, while the change between the second and third samples is attributed to the canagliflozin treatment. The laboratory reference range for each parameter is included. P-values, derived using the Wilcoxon Signed-Rank Test, provide a statistical comparison for the changes attributed to placebo versus those attributed to canagliflozin. To convert GLDH values from nkat/L to U/L, use a conversion factor of 0.06. For GGT values, the conversion factor from µkat/L to U/L is 60

| Parameter | Laboratory reference range | 1^st^ sample | 2^nd^ sample | 3^rd^ sample | Change placebo (2^nd^ sample – 1^rd^ sample) | Change canagliflozin  (3^rd^ sample –  2^nd^ sample) | p-value |
| --- | --- | --- | --- | --- | --- | --- | --- |
| GLDH (nkat/L) | 10-115 | 81.6  (80.1) | 88.8 (130.5) | 495.9 (591.3) | 2.6  [+3.1 %] | 357.0  [+402.0 %] | 0.055 |
| GGT (µkat/L) | 0.1-0.5 | 0.23  (0.033) | 0.24  (0.83) | 0.26 (0.128) | 0.02  [+6.5 %] | -0.02  [-6.3 %] | 0.2 |
| TG (mmol/L) | 0.1-0.5 | 0.27  (0.24) | 0.32  (0.06) | 0.40 (0.28) | 0.03  [+9.4 %] | 0.08  [+23.8 %] | 0.008* |
| Total protein (g/L) | 56-72 | 65.7  (2.8) | 69.4  (5.6) | 63.7  (1.9) | 3.1  [+4.7 %] | -5.9  [-8.5 %] | 0.008* |
| Albumin (g/L) | 29-37 | 33.2  (1.2) | 34.2  (1.8) | 31.7  (2.0) | 1.1  [+3.3 %] | -2.6  [-7.4 %] | 0.008* |
| SAA (mg/L) | <5 | <2 | <2 | <2 | NA | NA | NA |
| Creatinine (µmol/L) | 77-145 | 136.9 (17.1) | 119.1  (14.7) | 124.7 (13.6) | -20.2  [-14.8 %] | 7.6  [+6.4 %] | 0.008* |
| Na^+^ (mmol/L) | 133-145 | 136.7  (1.5) | 138.5  (4.3) | 137.1  (3.4) | 2.4  [+1.7 %] | -3.7  [-2.7 %] | 0.04* |
| Cl^-^ (mmol/L) | 97-108 | 101.4  (2.6) | 103.5  (1.2) | 100.1  (0.7) | 1.5  [+1.4 %] | -3.1  [- 3.0 %] | 0.02* |
| K^+^ (mmol/L) | 2.6-5.2 | 4.1  (0.4) | 3.8  (1.5) | 4.1  (0.5) | -0.5  [-11 %] | 0.3  [+7.4 %] | 0.3 |

* p<0.05

Abbreviations: GLDH, Glutamate dehydrogenase; GGT, Gamma-glutamyltransferase, TG, Triglycerides; SAA, Serum amyloid A
